# Supplementary material for: Discovery of small molecule inhibitors of Plasmodium falciparum apicoplast DNA polymerase
Source: J Enzyme Inhib Med Chem. 2022 May 5;37(1):1320–6. doi: 10.1080/14756366.2022.2070909 (PMC9090415; doi:10.1080/14756366.2022.2070909)
Supplement: Supplemental Material [file IENZ_A_2070909_SM3592.zip › supplemental figures.pptx]

## Slide 1
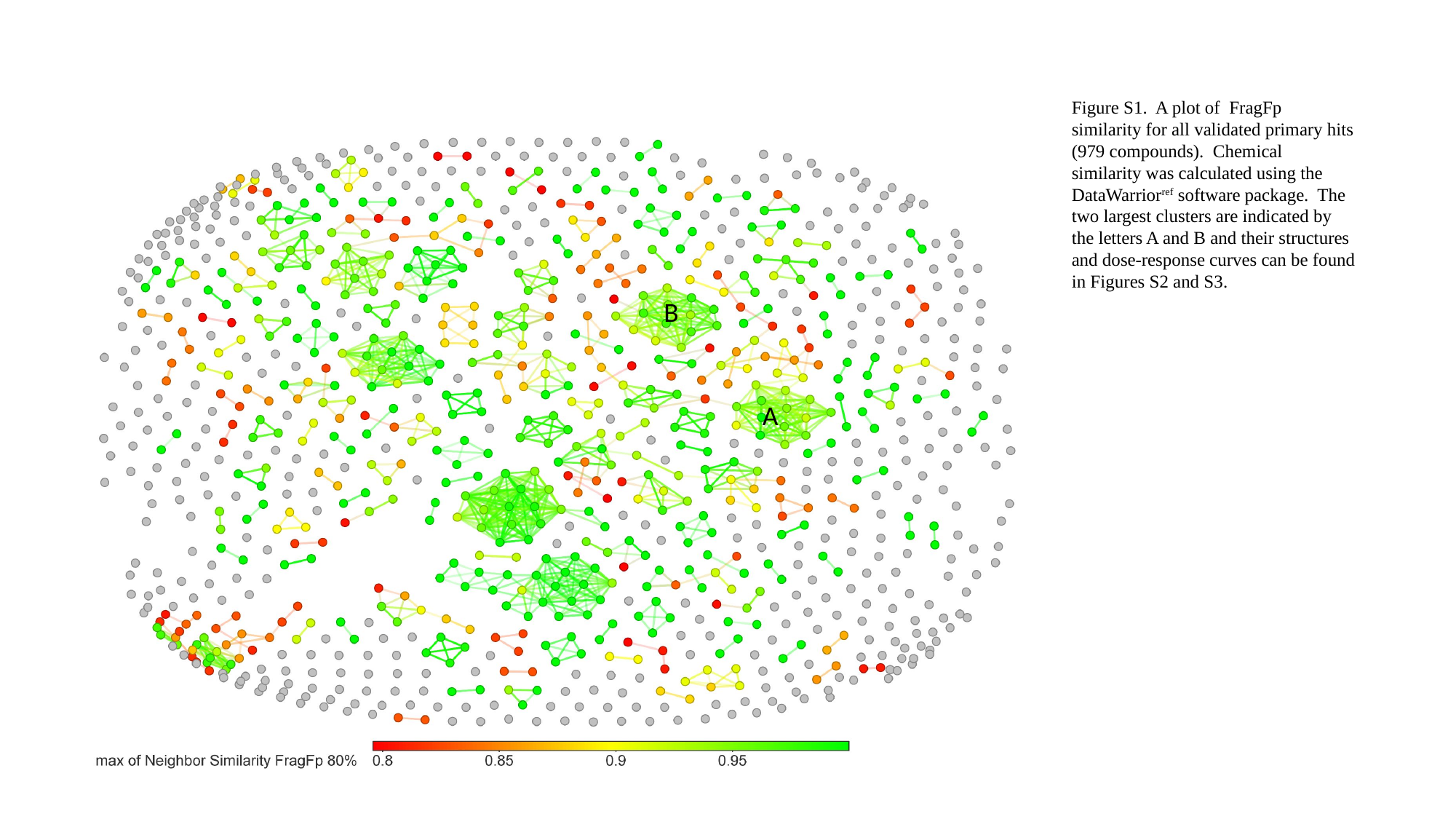

Figure S1. A plot of FragFp similarity for all validated primary hits (979 compounds). Chemical similarity was calculated using the DataWarriorref software package. The two largest clusters are indicated by the letters A and B and their structures and dose-response curves can be found in Figures S2 and S3.
B
A

## Slide 2
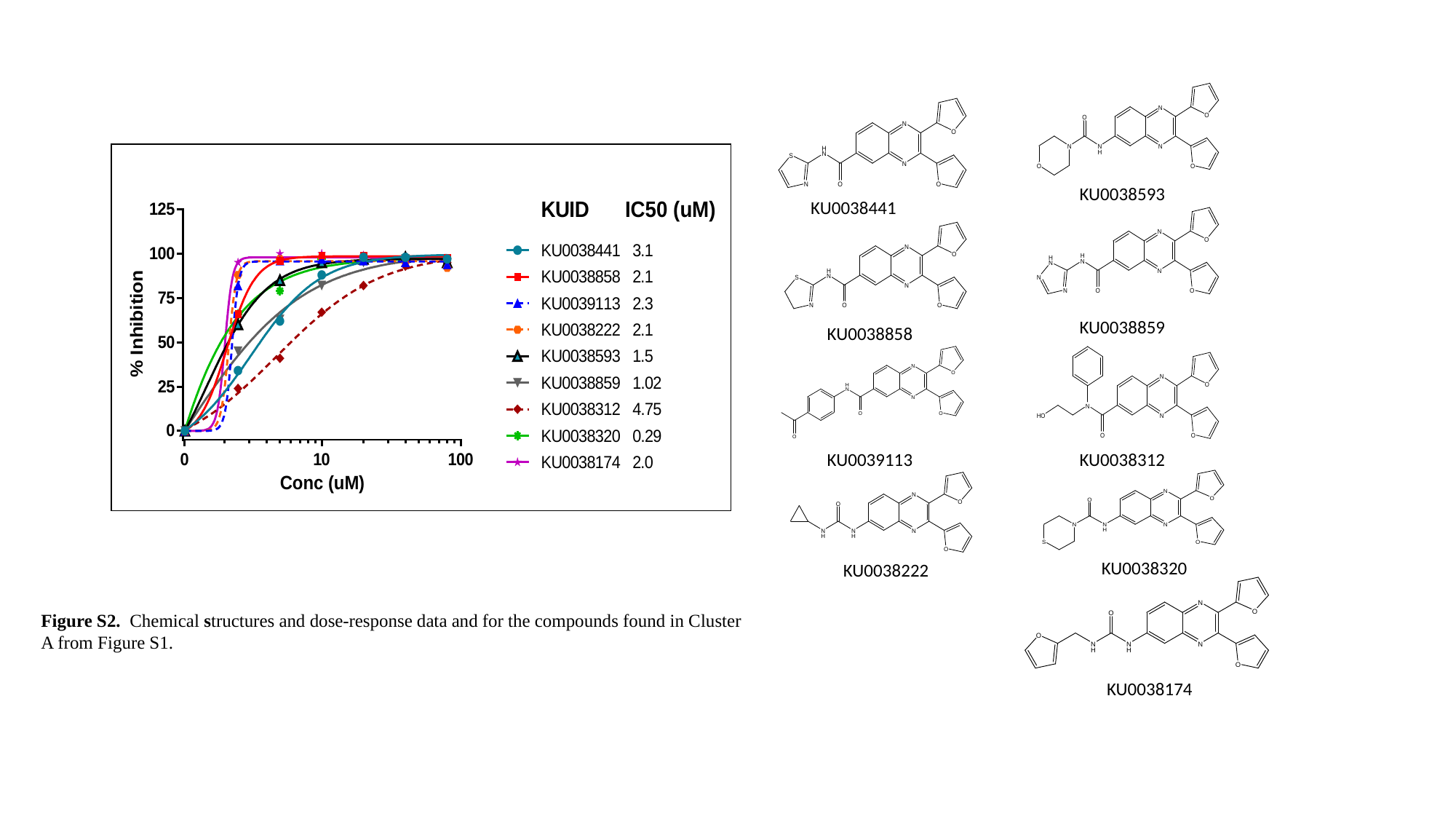

KU0038593
KU0038441
KU0038859
KU0038858
KU0039113
KU0038312
KU0038320
KU0038222
KU0038174
Figure S2. Chemical structures and dose-response data and for the compounds found in Cluster A from Figure S1.

## Slide 3
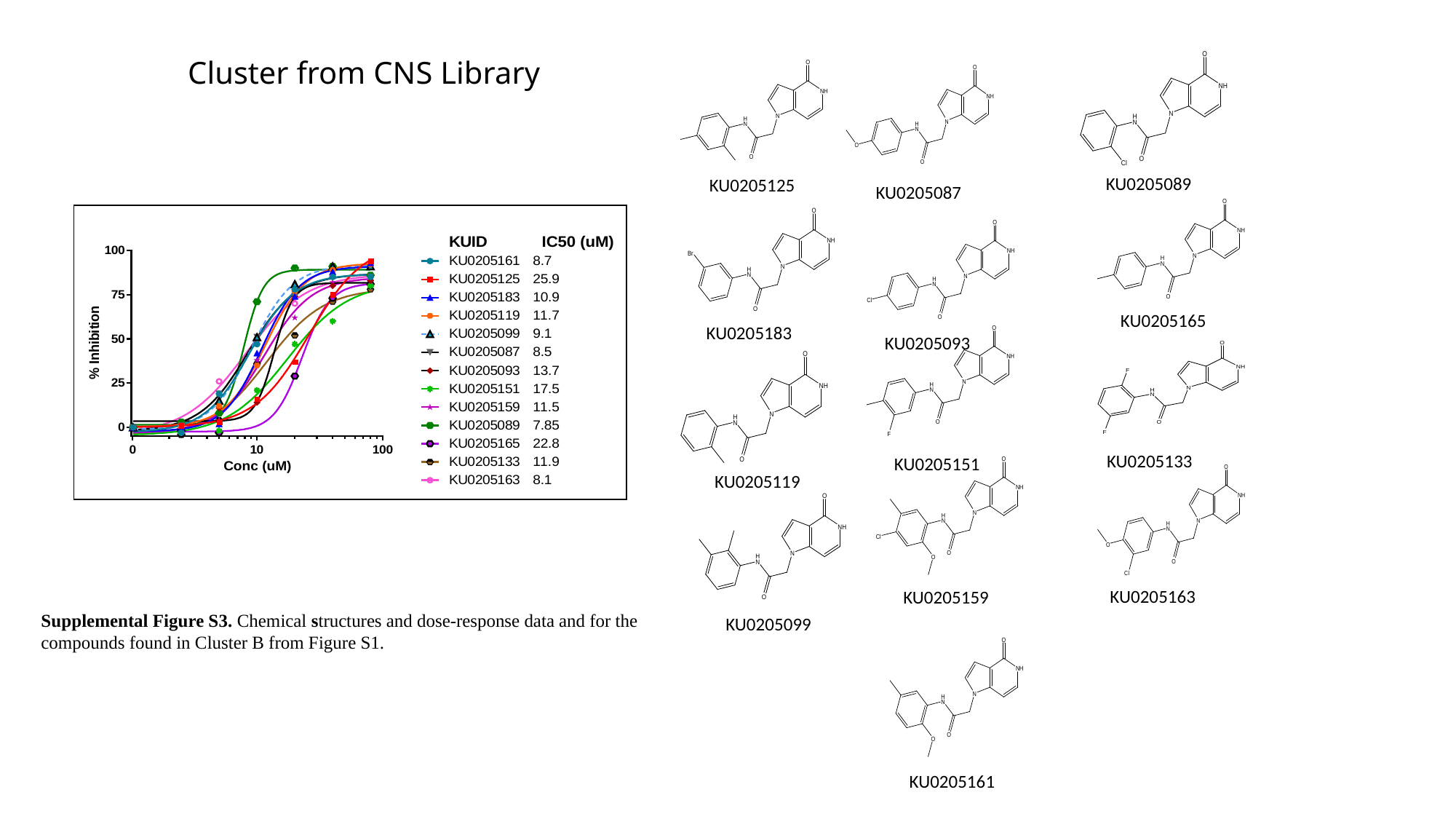

Cluster from CNS Library
KU0205089
KU0205125
KU0205087
KU0205165
KU0205183
KU0205093
KU0205151
KU0205133
KU0205119
KU0205159
KU0205163
KU0205099
Supplemental Figure S3. Chemical structures and dose-response data and for the compounds found in Cluster B from Figure S1.
KU0205161

## Slide 4
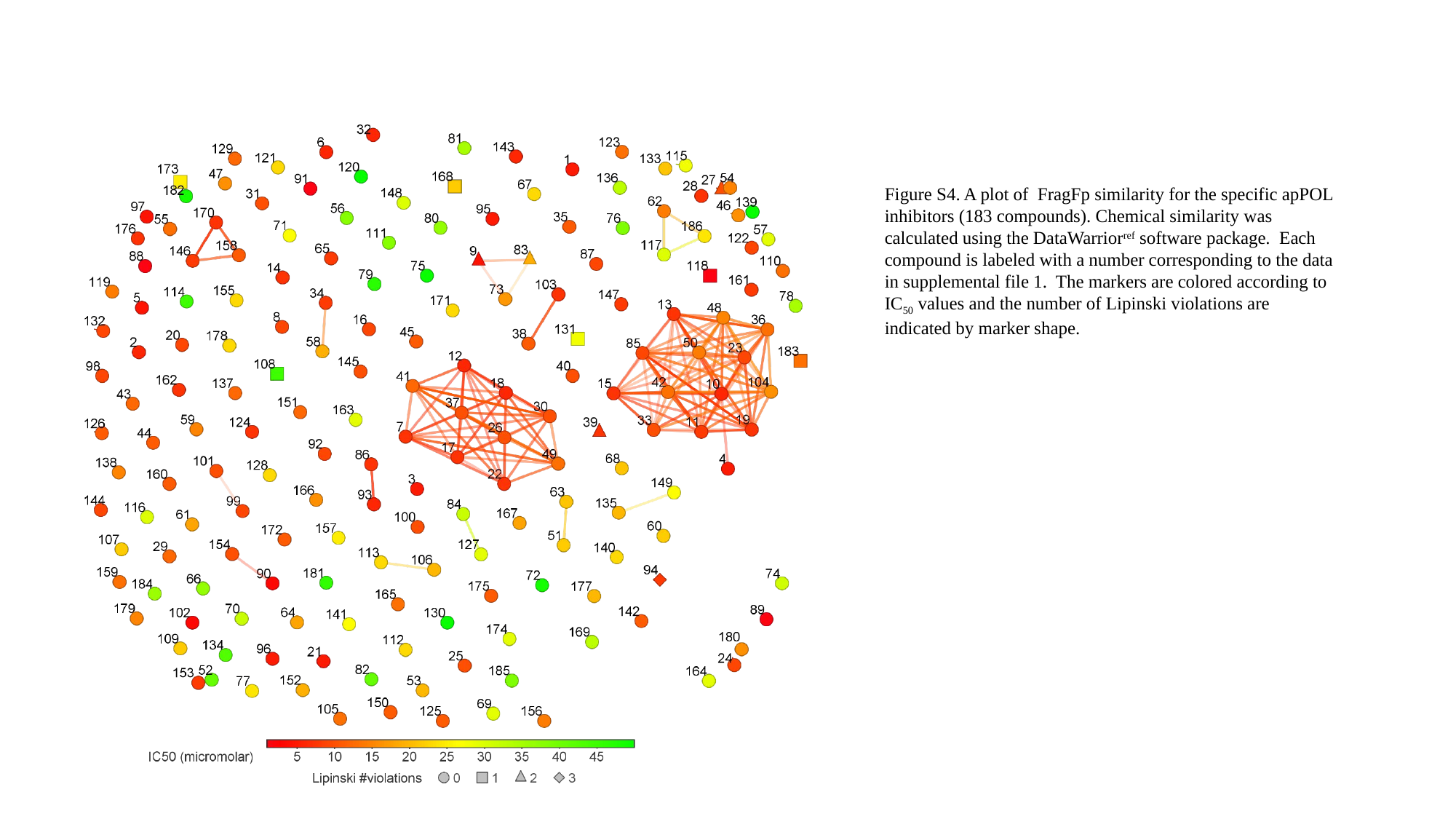

Figure S4. A plot of FragFp similarity for the specific apPOL inhibitors (183 compounds). Chemical similarity was calculated using the DataWarriorref software package. Each compound is labeled with a number corresponding to the data in supplemental file 1. The markers are colored according to IC50 values and the number of Lipinski violations are indicated by marker shape.
